# Highly Thermally Conductive Epoxy Composites with AlN/BN Hybrid Filler as Underfill Encapsulation Material for Electronic Packaging

**DOI:** 10.3390/polym14142950

**Published:** 2022-07-21

**Authors:** William Anderson Lee Sanchez, Jia-Wun Li, Hsien-Tang Chiu, Chih-Chia Cheng, Kuo-Chan Chiou, Tzong-Ming Lee, Chih-Wei Chiu

**Affiliations:** 1 Department of Materials Science and Engineering, National Taiwan University of Science and Technology, Taipei 10607, Taiwan; williaxom@gmail.com (W.A.L.S.); a12352335@gmail.com (J.-W.L.); hchiu@mail.ntust.edu.tw (H.-T.C.); 2 Graduate Institute of Applied Science and Technology, National Taiwan University of Science and Technology, Taipei 10607, Taiwan; cccheng@mail.ntust.edu.tw; 3 Material and Chemical Research Laboratories, Industrial Technology Research Institute, Hsinchu 31040, Taiwan; jeffreychiou@itri.org.tw (K.-C.C.); tzmlee@itri.org.tw (T.-M.L.)

**Keywords:** electronic encapsulation packaging, underfill material, hybrid filler, aluminum nitride, boron nitride, viscosity, thermal conductivity

## Abstract

In this study, the effects of a hybrid filler composed of zero-dimensional spherical AlN particles and two-dimensional BN flakes on the thermal conductivity of epoxy resin were studied. The thermal conductivity (TC) of the pristine epoxy matrix (EP) was 0.22 W/(m K), while the composite showed the TC of 10.18 W/(m K) at the 75 wt% AlN–BN hybrid filler loading, which is approximately a 46-fold increase. Moreover, various essential application properties were examined, such as the viscosity, cooling rate, coefficient of thermal expansion (CTE), morphology, and electrical properties. In particular, the AlN–BN/EP composite showed higher thermal stability and lower CTE (22.56 ppm/°C) than pure epoxy. Overall, the demonstrated outstanding thermal performance is appropriate for the production of electronic packaging materials, including next-generation flip-chip underfills.

## 1. Introduction

Technology has advanced significantly over time in response to the requirements of clients, both domestic and industrial [1]. As a result, electronic components have been developing into more advanced devices [2]. The increase in the processing speed of devices [3] such as computers and smartphones is one of the drastic improvements realized in recent decades. Nonetheless, these advancements have also resulted in the increase in heat flux from electronic components, owing to the increase in heat produced during the device operation [4,5,6,7]. It is well known from several studies and experience that the stability and lifespan of electronic components are affected directly by the thermal regime during their operation [8,9]. Consequently, it is essential that the heat accumulated in the device is dissipated as fast as possible to maintain the operating temperatures of devices at an admissible level. Recently, electronic encapsulation and the development of underfill materials have attracted considerable interest in heat dissipation and have become the most cutting-edge approaches in this field. An ideal underfill material must not only have high thermal conductivity (TC) but also low coefficient of thermal expansion (CTE), as underfilling shields the die bumps from thermomechanical stress generated between the die and substrate or another die, thus improving the link strength of electrical junctions and compensating for the differences in thermal expansion between two connecting materials, which might lead to device malfunction [10,11,12].

In most cases, underfill materials are epoxy-based composites with fillers that possess high TC but are electrically insulating; for instance, epoxy resin filled with silica particles. Although polymer composites show exceptional performance, underfill encapsulating materials with better mechanical properties and higher TC are frequently required for next-generation chip connections [13,14]. The addition of highly thermally conductive fillers has been widely employed to obtain composites with TCs higher than those of epoxy matrices, which are generally quite low (0.1–0.4 W/(m K)) [15,16]. Characteristic filler materials comprise metals (i.e., silver, aluminum, and copper) and ceramics (i.e., AlN and BN) [17,18]. However, with the rapid progress in materials and technology, diverse novel materials such as carbon-based materials (i.e., carbon fibers, carbon nanotubes, and graphene) have been employed to improve the encapsulant TC [19,20]. However, the high electrical conductivity of carbon nanomaterials, high cost, and assembly line production restrict the wide usage of such novel materials in electronic packaging [21,22,23]. A high filler content (>50 wt%) is required to achieve superior TC of the underfill material when using conventional micro-sized spherical particles or platelets. Moreover, poor flowability and the high costs of the material are typically the results of the particularly high filler content, limiting the practical use of electronic encapsulation packaging [24,25]. AlN has attracted considerable interest because of its high intrinsic TC (285–320 W/(m K)), high electrical insulation, and, in particular, its spherical particle shape [26,27,28], which enables simple processability and makes extremely large filler content (>60 wt%) feasible. Similarly, bulk hexagonal BN (h-BN) has a remarkably high in-plane TC (250–400 W/(m K)) [29,30,31,32], which is expected to be in considerable demand for underfill applications.

Nevertheless, numerous studies have shown that it is hard to obtain the aforementioned TC by enhancing dispersibility and achieving a high BN filler content because of the inherent features of flake-like particles [33,34,35]. TC greatly depends on the links between filler particles, namely thermal conduction networks. The construction of these conductive pathways is affected by various factors, such as filler loading [36], filler dispersion [37], filler intrinsic attributes (i.e., size, geometry, type, and TC) [38], filler surface functionalization [39], filler alignment [40], filler–matrix interface compatibility [41], and processing conditions [42]. At the same time, the development of continual thermally-conductive channels in the polymer matrix by combining various material properties and constructing a 3D interconnected heat-conductive structure is a favorable approach to improving filler utilization, reducing the matrix–filler interface thermal resistance and increasing the TC. Several studies have reported higher TCs by employing hybrid fillers with various dimensions in composites. Huang et al. [43] attributed the enhanced TC to the beneficial structure of h-BN-reduced graphene oxide and excellent hybrid filler dispersion in the epoxy matrix (EP). Feng et al. [44] prepared high-density polyethylene with 30 wt% BN and 3 wt% multi-walled carbon nanotubes as fillers. In this study, the ternary composites showed a higher TC (1.54 W/(m K)) than the binary composites (1.19 W/(m K)). Thus, Feng et al. [44] concluded that the synergistic effect of the fillers endowed the ternary composites with superior TC. Liu et al. [45] made polydimethylsiloxane composites with aligned BN and Al_2_O_3_ as a hybrid filler by 3-D printing; they ascribed the improved TC to the synergistic effect of the hybrid fillers, which reduced the thermal interface resistance. The above discussion stresses the advantages of employing hybrid fillers: they increase the probability of forming more thermal connections between the polymer and filler interface, building a 3D heat-conductive network, and thus boosting the TC. Nevertheless, the heat conduction resistance between the polymer and filler interface may occur because of structural differences. According to the literature, surface modification can successfully enhance the filler–matrix interaction while decreasing the interfacial thermal resistance [46,47]. As a result, minimizing the indicated resistance can result in composites with superior TC and other properties, as can be found in the literature. Bekeshev et al. [48] prove that epoxy composites with ocher as filler can increase its physical and mechanical properties by the utilization of microwave modification with optimal parameters (power of 350 W and duration of 30 s), which increased the composite toughness by 18%, tensile strength by 29% and bending strength by 26%. Likewise, Mostovoy et al. [49] showed that the functionalization of MWCNTs with γ-aminopropyltriethoxysilane and the creation of chemical bonds in the polymer–filler interface were responsible for the improvements in the physicomechanical properties of the composite, such as increased impact strength by 300%, bending stress by 194%, bending modulus by 137%, tensile strength by 108% and tensile elastic modulus by 52%. Furthermore, integrated circuit (IC) underfill materials must have high electrical resistivity to avoid internal disturbance or damage to the device electronic system, and low relative permittivity to maintain quick signal transmission. According to the literature, these properties are mostly affected by the intrinsic filler characteristics, as well as the applied frequency. Huang et al. [50] indicated that the dielectric permittivity variation in epoxy composites is affected by both the high dielectric constant of h-BN and graphene oxide, as well as the frequency. Similarly, Jiang et al. [51] reported that the EP/h-BN-poly(glycidyl methacrylate) composite exhibited excellent dielectric characteristics, owing to the system frequency and natural attributes of the fillers.

In this study, a simple approach was used for the fabrication of potential electronic underfill materials. The viscosity of composites was considered the decisive characteristic for establishing the most promising composition of the hybrid filler, consisting of zero-dimensional spherical AlN and two-dimensional BN flakes. The electric properties, TC, coefficient of thermal expansion, cooling rate, decomposition temperature, glass transition temperature, and morphology of the composites were analyzed to study the effects of the hybrid filler on the properties of the pristine epoxy.

## 2. Experimental

### 2.1. Materials

**Figure 1:**
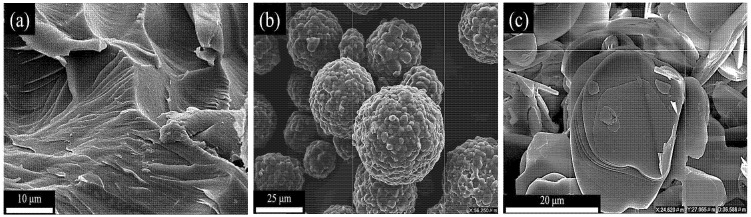
SEM images of (**a**) EP, (**b**) AlN spherical particles, and (**c**) BN platelets.

Bisphenol-F epoxy resin (EXA-830LVP) was supplied by the DIC Company, Japan. Aromatic amine (KAYAHARD A-A; Nippon Kayaku Co., Ltd., Tokyo, Japan) and silane (Xiameter OFS-6040; Dow Inc., Midland, TX, USA) were used as the curing and coupling agents, respectively. Spherical AlN particles (97%) were purchased from Thrutek Company, Taiwan. Flake-like hexagonal boron nitride (BN, 99%) powder was supplied by King Meitek Industrial Co., Ltd., New Taipei City, Taiwan. All chemicals were used as obtained, without further treatment. Additional information regarding the epoxy and fillers is summarized in Table 1 and the micrographs of the EP cross section and filler morphologies are presented in Figure 1.

### 2.2. Selection of Epoxy Binder System

The EP consisted of epoxy resin, a curing agent, and silane. First, this research studied distinct types of epoxy resin systems by incorporating various types of epoxy resin, curing agent, and silane. Initially, the epoxy resin and curing agent were mixed at a weight ratio of 1:0.8 using a planetary centrifugal mixer (THINKY mixer) at 2000 rpm for 20 min. Subsequently, 1.5 wt% of silane was added to the obtained mixture and further blended in the mixer. After preparation, the viscosity of each epoxy resin system was determined. Finally, based mainly on the lowest measured viscosity, an epoxy resin system was chosen.

### 2.3. Preparation of AlN–BN/EP Composites

**Figure 2:**
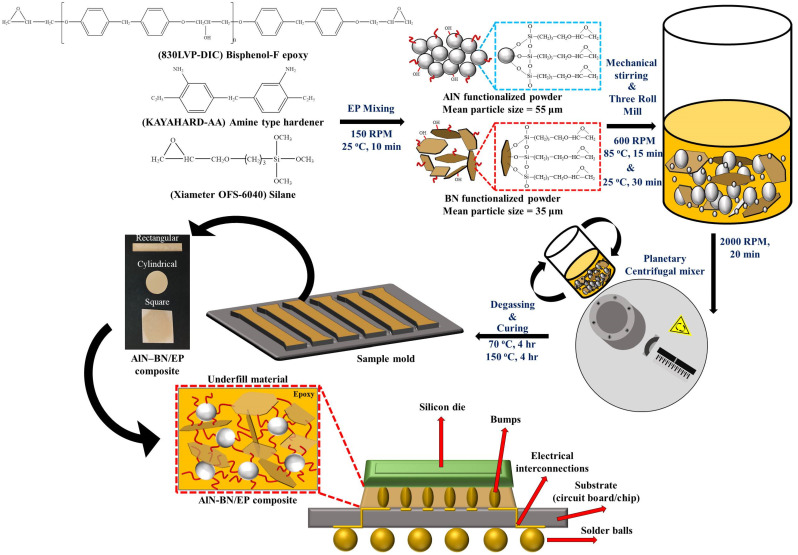
Schematic of the preparation of AlN–BN/EP composites.

The schematic of the fabrication of the AlN–BN/EP composites is shown in Figure 2, along with the location of the material in the devices in which it is applied. First, for the procedure of surface modification of fillers, all fillers were preheated for 12 h at 100 °C to eliminate moisture. Subsequently, the fillers and NaOH were blended at 120 °C for 24 h; next, the solution was washed multiple times with deionized water and filtered; then, the solution was dried in an oven at 80 °C for 24 h; finally, the AlN- and BN-functionalized fillers were obtained and later stored in a chamber with controlled humidity for future use. For the epoxy composites fabrication procedure, first, only the pristine epoxy was pre-heated for 6 h at 90 °C to melt the crystals that were formed at room temperature. Subsequently, EP was put into a beaker and blended for 10 min at 150 rpm and room temperature. Afterwards, the BN- and AlN-functionalized powders were placed in the previous mixture at various filler loadings, followed by hand mixing for 5 min. Thereafter, the preceding mixture was mixed for 10–20 min using a mechanical stirrer at 600 rpm and 70–100 °C; next, the filler particles were dispersed in the EP matrix using a three-roll miller for 20–30 min. Later, it was stirred once again in the THINKY mixer at 2000 rpm for 20 min to homogenize the blend. Subsequently, the final solution was transferred to a Teflon mold and placed in a vacuum oven for degassing at 70–130 °C for 1–4 h, depending on the composite viscosity. The viscosity of the obtained solution was measured. Finally, the sample was cured at 150 °C for 4–5 h, and AlN–BN/EP composite was obtained. The same procedures were used to prepare the remaining epoxy resin composites, denoted as EP (without filler), AlN/EP (with spherical AlN filler), BN/EP (with BN flake-like filler), and AlN–BN/EP (with AlN and BN hybrid filler).

### 2.4. Characterization and Instruments

Fourier transform infrared (FT-IR; FT/IR-4600, JASCO, Tokyo, Japan) spectra of the composite samples were obtained in the range 4000–500 cm^−1^ with pure KBr as the background. The viscosities of the pre-cured AlN–BN/EP composites were estimated using a modular compact rheometer (MCR 102, Anton-Paar, Graz, Austria) at 30–35 °C and a constant shear rate of 10 s^−1^. The composite viscosities were measured promptly after the centrifugal mixing stage of the sample preparation, as indicated in Section 2.3. The TCs of the samples were determined using a thermal constant analyzer (TPS 2500 S, Hot Disk, Gothenburg, Sweden) at room temperature; the mean of five measurements was acquired for each sample. The samples were cylindrical, as mentioned in Section 3.3. The heat transfer capability of the EP composites was evaluated using (FLIR ONE Pro-iOS) a thermographic camera at 30–35 °C. The linear CTE of the cured AlN–BN/EP composites was measured using a thermal mechanical analyzer (TMA Q400, TA instruments, New Castle, DE, USA). The samples were heated in an analyzer furnace at a ramp rate of 5 °C/min from room temperature to 130 °C under nitrogen atmosphere. Samples for scanning electron microscopy (SEM) were prepared by shattering a rectangular sample using diagonal cutting pliers and coating the sample surface with platinum. The morphology of all composites was characterized using a field emission scanning electron microscope (JSM-6500F, JEOL, Tokyo, Japan). The thermal stability of EP composites was confirmed using thermogravimetric analysis (TGA; Q500, TA Instruments, USA) in a nitrogen atmosphere over the temperature range of 35–700 °C at a heating rate of 10 °C/min. Dielectric properties were measured using a precision impedance analyzer (8722ES, Agilent, Santa Clara, CA, USA) at room temperature over the frequency range of 10^2^–10^6^ Hz. Samples with a square shape were made to carry out the test, as indicated in Section 3.3, the 85052B electrode sensor was utilized, and the applied voltage was based on the built-in voltage of the analyzer.

## 3. Results and Discussion

### 3.1. FTIR Analysis of EP Composites

**Figure 3:**
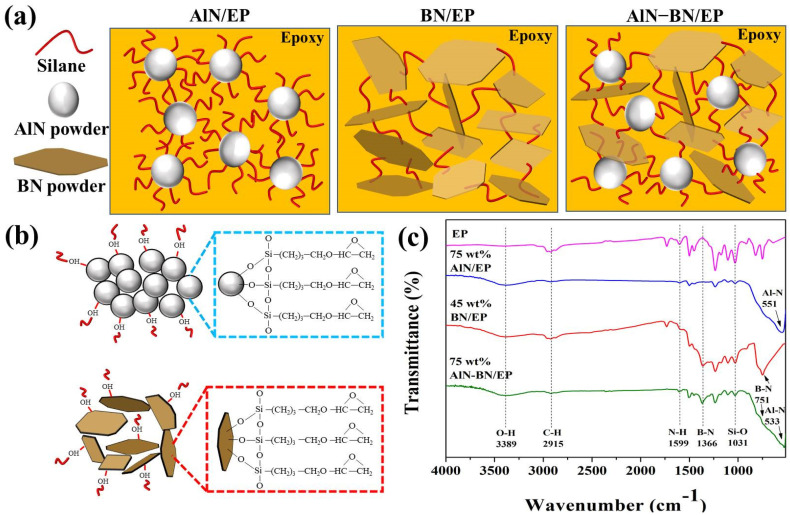
(**a**) Schematic of the particle arrangement in the epoxy matrix, (**b**) Diagram of the mechanism of the modification of AlN and BN filler surface with EP, and (**c**) FTIR curves of the EP composites.

Figure 3a illustrates the arrangement of different fillers within the epoxy matrix, Figure 3b shows the reaction between the functionalized fillers and EP, and Figure 3c displays the FTIR spectra of EP and different composites prepared at the highest feasible filler loading achieved in this study. The wide absorption band observed at approximately 551 cm^−1^ on AlN/EP was ascribed to the Al–N stretching vibration [52,53,54], and the broad peak at approximately 533 cm^−1^ for the AlN–BN/EP composite. Furthermore, in the curves of BN/EP and AlN–BN/EP, two prominent absorption peaks at approximately 751 and 1366 cm^−1^ were assigned to the out-of-plane bending vibration of B–N–B and in-plane stretching vibration of B–N, respectively [55,56,57,58]. The peaks observed in the spectra of all EP composites at 1031 cm^−1^ were attributed to the Si–O vibration [59,60,61]. Although this peak is quite intense in the spectrum of EP, it diminishes with an increase in filler loading because of a reduction in the silane volume in the composite mixture. In addition, a wide absorption band is observed around 3389 cm^−1^ for the as-prepared composites, which is characteristic of the stretching vibration of O–H [57,58,59,62]. This peak may be ascribed to the O–H on the silane and H_2_O molecules adsorbed on the filler surface from the atmosphere. These results indicate the presence of silane, AlN, and BN fillers in the prepared composites.

### 3.2. Rheological Study and TC of EP Composites

**Figure 4:**
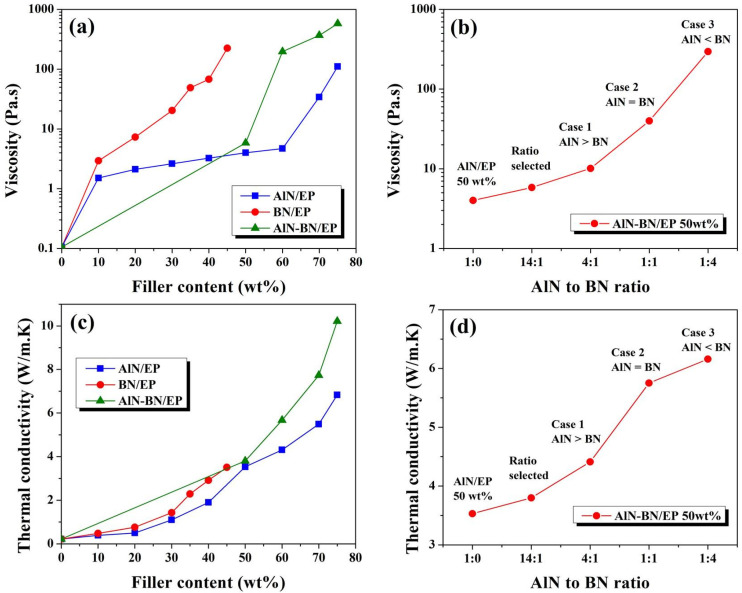
(**a**) Viscosity graphs of (**a**) EP composites versus filler loading and (**b**) AlN–BN/EP at a fixed total filler content of 50 wt% with different ratios of AlN and BN particles. TC of (**c**) EP composites versus filler loading and (**d**) AlN–BN/EP at a fixed total filler content of 50 wt% with different ratios of AlN and BN particles.

This study primarily focused on analyzing the rheological behavior of various composites with a single filler to determine the most favorable proportions of AlN and BN fillers in a hybrid filler, as mentioned in Section 2.2. The viscosity of EP composites at different filler loadings is shown in Figure 4a. The viscosity of pristine EP is 0.103 Pa s, and the viscosities of the composites at the highest filler content obtained in this study are as follows: AlN/EP 75 wt% (111 Pa s), BN/EP 45 wt% (225 Pa s), and AlN–BN/EP 75 wt% (579 Pa s). According to the results, the viscosity is affected by the particle shape, particle size, and filler loading [63,64]. At small filler loadings, the viscosity of BN/EP rapidly increases with filler loading, which is attributed to the geometric nature of BN particles. AlN/EP composites show considerably lower viscosity than BN/EP composites over the entire range of filler contents, which was ascribed to the larger size and spherical shape of the particles in AlN, enabling easier polymer flow. At the same time, the viscosities of the AlN–BN/EP composites are higher than those of the AlN/EP composites over the entire range of filler loading, as a result of the incorporation of BN particles into the mixture, thereby resulting in stronger polymer–filler interactions. Figure 4b shows the viscosity of AlN–BN/EP composites at a fixed total filler loading of 50 wt% but different ratios of AlN to BN. A filler loading of 50 wt% was chosen based on the viscosity results acquired for (AlN/EP) because, above this filler amount, the viscosity significantly increases, as shown in Figure 4a; thereby, this value was attributed to the composite percolation threshold [65,66,67]. As illustrated in Figure 4b, three cases were proposed to determine the optimal ratio of the hybrid filler: Case 1 (AlN wt% > BN wt%), Case 2 (AlN wt% = BN wt%), and Case 3 (AlN wt% < BN wt%). In Figure 4b, with a further increase in the BN filler loading, the viscosity of the hybrid filler composed of AlN/EP and BN/EP increases significantly. Thus, the ratio between the origin of the curve (AlN/EP 50 wt% without BN filler) and Case 1 was chosen because the hybrid filler ratios in this area of the curve resulted in the smallest viscosity values of this specific hybrid composite. A ratio of 14:1 was chosen, and it was employed for producing AlN–BN/EP composites with various filler contents. Further comparisons of this composite system with those with different component ratios are presented in various sections of this paper. Figure 4c illustrates the changes in the TC of the distinct EP composites at different filler loadings. EP exhibits an extremely low TC of 0.22 W/(m K). Overall, TC increases with filler content in all cases, which may be attributed to the fact that as the filler content increases, more AlN particles interact with each other, creating efficient heat conductive channels and increasing thermal diffusion through the composite; additionally, it was noticed that the TC is affected by the geometry of filler particles [39,40,56,66,67,68,69,70,71]. The TC of the BN/EP composites is slightly higher than that of AlN/EP composites at lower filler loadings. For instance, at 30 wt% filler loading, the TC of BN/EP is 1.43 W/(m K), while that of AlN/EP is 1.10 W/(m K). This difference was ascribed to the exceptional TC of BN particles, which is slightly higher than that of AlN particles. However, the addition of BN filler content is limited to 45 wt% because BN has flake-like particles, which possess a higher surface area than AlN spherical-like particles, thereby restricting epoxy polymer chain motion. Meanwhile, the AlN ball-shaped particles in AlN/EP allowed the composite to reach a higher filler content due to the smaller contact area between the particles; therefore, they could be dispersed better within the polymer matrix and allow the polymer to flow easily. The maximum TC obtained in this study is 10.18 W/(m K) for the AlN–BN/EP composite at 75 wt% of filler loading, which is approximately 46 times higher than that of EP and approximately 49% higher than that of the AlN/EP 75 wt% (6.83 W/(m K)). This result was attributed to the incorporation of a small amount of BN particles, which, in combination, built a 3D interconnected heat-conductive structure with a more effective arrangement of thermal conduction networks [43,44,45,46,47]. Figure 4d shows the TC of AlN–BN/EP composites at a fixed total filler loading of 50 wt% with different ratios of AlN to BN. The obtained results suggest that with a further increase in BN filler loading, the TC of the AlN–BN/EP composite also considerably increased; however, at this filler loading, the BN filler loading increases the TC at the cost of exponential growth in the viscosity of the composite, as noted from the results indicated in Figure 4b. As a result, this hinders the achievement of a superior packing density in the polymer matrix, thus restricting the filler capacity of the composite and ultimately limiting the potential for achieving a higher TC. The TC achieved in this study is comparable to that reported in recent publications on AlN and BN composites. Table 2 provides a detailed comparison of these findings. These results suggest that the AlN/BN hybrid filler can significantly improve the TC of epoxy composites.

### 3.3. Thermal Management Capability of Composites

**Figure 5:**
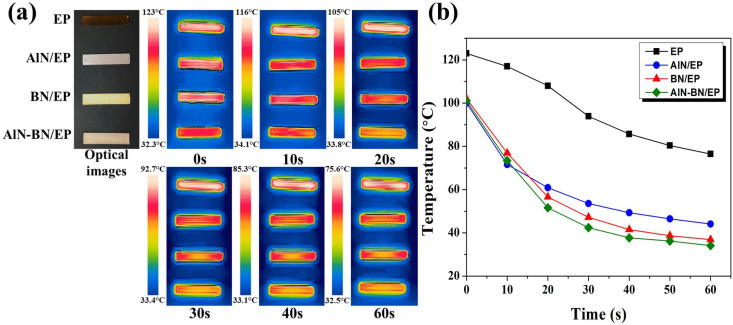
(**a**) Optical photographs and infrared thermal images of the as-prepared composites and (**b**) cooling curves of the composites and pristine EP.

**Figure 6:**
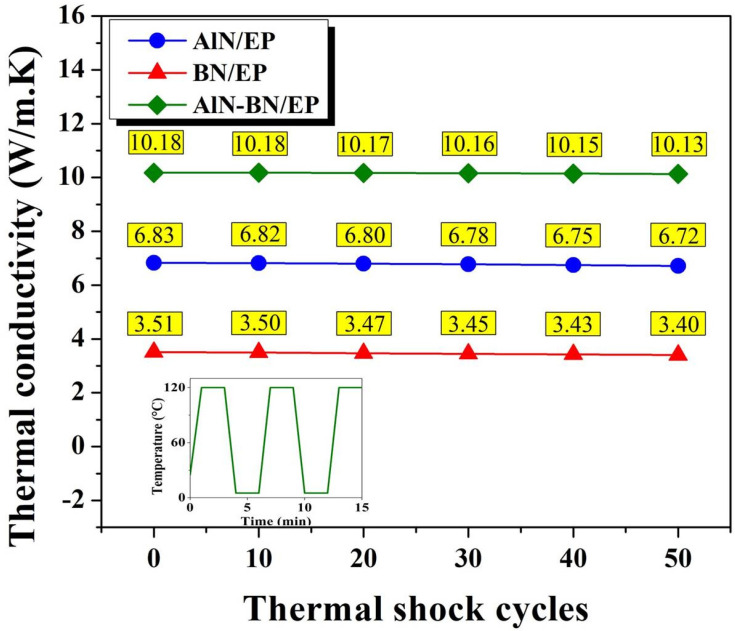
Resistance to thermal shocks of the EP composites.

Figure 5a and Video S1 in the Supporting Information show the optical and infrared thermal images of rectangular-shaped pristine EP and composite samples, and Figure 5b illustrates the change in the temperature of EP composites with time during cooling. The pristine EP exhibits a dark yellow color, the AlN/EP exhibits a light gray color, the BN/EP shows a vanilla color, and the AlN–BN/EP exhibits an ecru color. Moreover, three following sample shapes were prepared for the distinct analyses described in this study: rectangular (84 mm in length, 13 mm in width, and ~2.5 mm in thickness), cylindrical (8.5 mm in height and 34 mm in diameter), and square (49 mm length, 49 mm width, and ~0.9 mm thickness). To assess the heat transfer capability of the EP composites, their cooling curves were recorded using a thermographic camera [18,40,44,47,56]. The composites at the highest filler loading obtained in this study were assessed using this method; all samples were preheated in an oven at 120 °C for 4 h. As displayed in Figure 5a,b, the single-filler composites show a faster heat sink capability than the pristine EP from the very beginning of the test. In addition, the AlN–BN/EP composite exhibits a much greater heat dissipation potential than the other three samples, which was ascribed to its higher TC, and consequently, a quicker heat-transfer response. For example, at 60 s, the AlN–BN/EP composite surface temperature is 34.1 °C, while those of BN/EP, AlN/EP, and EP are 36.9 °C, 44.1 °C, and 76.5 °C, respectively. Additionally, the reliability of the composites applied under severe conditions is vital; accordingly, an accelerated aging test of the EP composites under thermal shock was carried out in two separated climatic chamber, high temperature chamber (120 °C) and low temperature chamber (5 °C), respectively. The specimens stayed in each chamber for 2 min alternately and the entire test was performed for 50 cycles. As illustrated in Figure 6, the TC of BN/EP is decreasing from 3.51 W/(m K) to 3.40 W/(m K) throughout the 50 cycles of thermal shocks, showing a TC reduction of 3.13%. Similarly, the AlN/EP composite presented a TC decline of 1.61% (from 6.83 W/(m K) to 6.72 W/(m K)), meanwhile the AlN–BN/EP composite exhibited a TC decrease of 0.49% (from 10.18 W/(m K) to 10.13 W/(m K)). The slight variation in TC may be ascribed to the CTE difference of AlN, BN and epoxy matrix, leading to interface discrepancy of the aforementioned materials [76]. As shown by the remarkable TC values before and after severe temperature variations, the hybrid filler composites have exceptional heat transfer ability, making them promising next-generation IC underfill materials that are effective at intense temperature changes during device operation. 

### 3.4. Impact of Fillers on T_g_ and CTE of Composites

**Figure 7:**
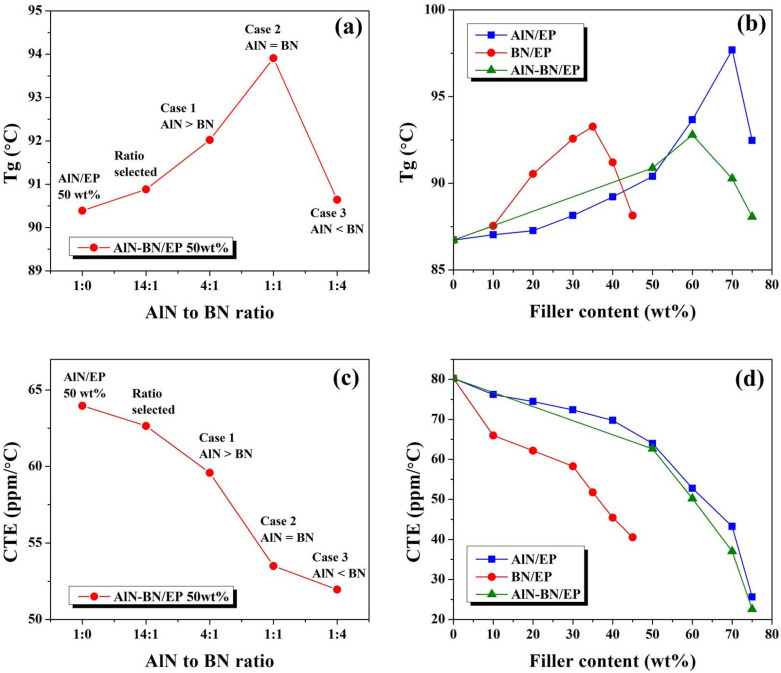
T_g_ graphs for (**a**) AlN–BN/EP composites at a fixed total filler content of 50 wt% with different ratios of AlN to BN particles and for (**b**) different EP composites versus filler loading. CTE below T_g_ graphs for (**c**) AlN–BN/EP composites at a fixed total filler content of 50 wt% with different ratios of AlN to BN particles and for (**d**) EP composites versus filler loading.

The glass transition temperatures (T_g_) of the EP composites are shown in Figure 7a,b. The glass transition temperature of pristine EP is 86.64 °C. Figure 7a displays the T_g_ of AlN–BN/EP composites at a fixed total filler loading of 50 wt% with different ratios of AlN to BN. The experimental findings suggest that the hybrid filler ratio is a key factor for the T_g_ value. For this specific composite, with increasing BN filler loading, the T_g_ increases until the AlN:BN = 1, after which it considerably declines, ultimately reaching a slightly higher value (90.63 °C) than that of pristine EP. Figure 7b presents the T_g_ of the EP composites versus the filler content. The chart shows that the composite T_g_ is affected by the filler loading and inherent geometry of the fillers [24,29,64,77,78]. In the BN/EP composite, increasing BN platelet loading in the polymer increases the T_g_ until 35 wt% BN and reduces it at higher filler contents, ultimately (at the maximum BN content of 45 wt%) resulting in a value (88.14 °C) slightly higher than that of EP. Several studies [77,78,79,80,81] reported that fillers with large surface areas show stronger interfacial interactions with the polymer matrix, resulting in changes in the crosslinking density and therefore a decrease in T_g_. The AlN/EP and AlN–BN/EP composites exhibit behavior similar to that of BN/EP. On the one hand, an increase in T_g_ indicates that the epoxy–filler interaction can effectively control the polymer chain mobility, and that this interaction can be improved through the increase in filler loading and optimum filler ratio distribution. This result can also be ascribed to the remarkable TCs of the EP composites. In particular, the greater the TC of the composite, the less heat is retained by the composite. As a result, the vibration of the polymer molecules in the composites is minimized. On the other hand, the T_g_ values of the composites decline at a very high filler content (~70 wt%), reaching 92.47 °C for AlN/EP and 88.06 °C for AlN–BN/EP, which was attributed to the increase in viscosity [78,79,81]. The increasing viscosity complicates the removal of the interstice air gap on the polymer–filler interface; as a result, the interaction between the filler and the matrix is weakened, making it challenging to successfully control the epoxy chain mobility. As shown in Figure 7c,d, the increase in the filler loading of either of the fillers results in a considerable decrease in the CTE of EP, which was attributed to the increasing TC of the composites and inherent CTE values of AlN and BN, which are lower than that of EP (80.32 ppm/°C). The AlN–BN/EP composite at 75 wt% exhibits the lowest CTE (22.56 ppm/°C), which is beneficial for maintaining dimensional stability. Numerous studies have reported that the CTE of composites is affected by the filler loading and inherent geometry of the filler [13,24,64,77,79,81,82]. For instance, the CTE of the BN/EP composite at 45 wt% is lower than those of AlN/EP and AlN–BN/EP at the same filler content, which was ascribed to the slightly lower CTE of BN compared to AlN and to the larger surface area of the BN flakes, resulting in a larger interfacial area between the filler and epoxy, and, consequently, limiting the thermal expansion of the polymer matrix. Likewise, the CTE significantly decreases for the AlN/EP and AlN–BN/EP composites at a very large filler content (~70 wt%), which could be ascribed to two factors. First, the viscosity of the composite exponentially increases at an extremely high filler content, exerting substantial mechanical and physical limitations on the polymer matrix. Second, the synergistic effect of the hybrid filler potentially results in a more efficient polymer–filler interaction, restricting the thermal expansion of the epoxy matrix [13,77,81,82].

### 3.5. Morphological Analysis of the EP Composites

**Figure 8:**
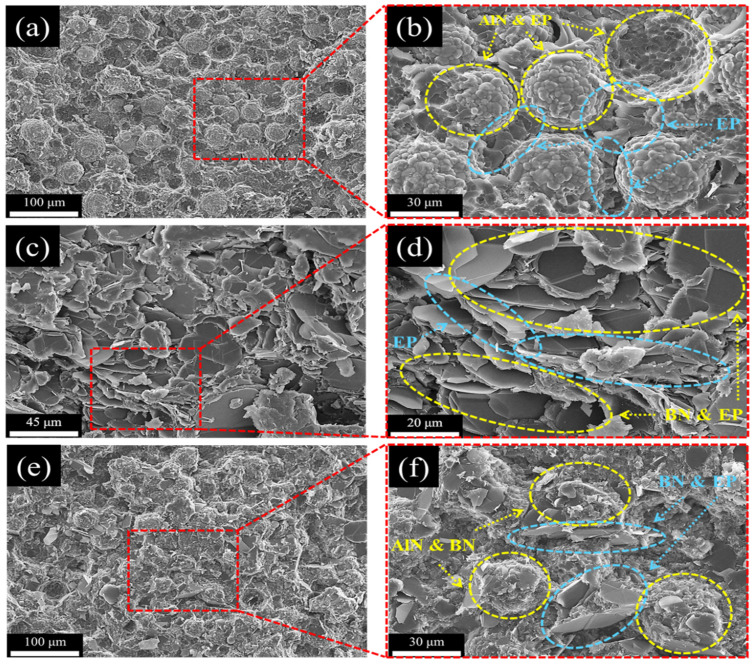
SEM micrographs of (**a**,**b**) the AlN/EP composite at 75 wt%, (**c**,**d**) BN/EP composite at 45 wt%, and (**e**,**f**) AlN–BN/EP composite at 75 wt%.

Cross-sectional SEM images of AlN/EP, BN/EP, and AlN–BN/EP are shown in Figure 8. As illustrated in Figure 8a,b, the AlN filler is very well distributed throughout the polymer matrix, which is attributed to the spherical shape of the AlN particles. However, spherical particles possess the least contact area; as a result, they do not form many efficient heat-conductive channels, even at high AlN filler loadings (75 wt%). Moreover, it can be observed in Figure 8c,d that the BN platelets are well dispersed and form thermally conductive paths; however, there is some agglomeration, which may be ascribed to two reasons. First, the inherent geometry of BN platelets, which possess a greater surface area, enables reciprocal adhesion of the particles. Second, the BN platelets are smaller than the AlN particles; as a result, the BN particles have a larger specific surface area, thereby intensifying the intermolecular forces that promote agglomeration. It can be inferred from the above discussion that because there is a substantial epoxy matrix layer between the filler particles, obtaining an impeccable heat-conductive pathway is challenging. Nonetheless, as observed in Figure 8e,f, there is a continuous connection with minor agglomeration in the AlN–BN/EP composite, suggesting that the AlN/BN hybrid filler forms a more effective interfacial linkage compared to the single fillers. The two-dimensional BN flakes have a smaller particle size than the zero-dimensional spherical-like AlN particles. As a result, the BN flakes can occupy the gaps between AlN particles through shear motion during mixing. This minimizes aggregation and results in the formation of a 3D heat-conductive network via thermal channels between the filler particles. Therefore, more functional heat pathways were generated in the AlN–BN/EP composites.

### 3.6. Thermal Stability of the Composites

**Figure 9:**
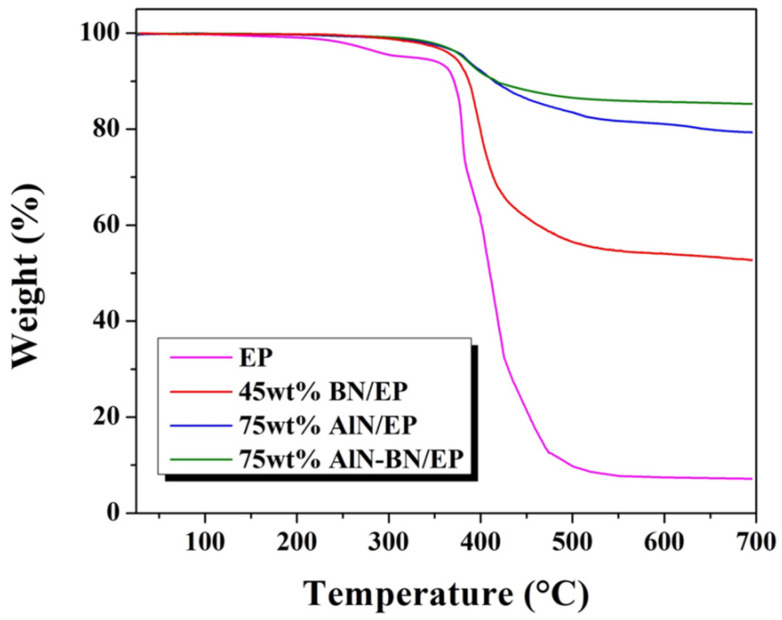
TGA graphs of pristine EP and the EP composites.

The TGA graphs of pristine EP and EP composites at the highest studied filler loading are shown in Figure 9. A minor weight loss is observed in pristine EP, starting at approximately 232.9 °C. A sharp decrease in the curve at 352.6 °C is correlated to the thermal decomposition of EP (T_d_), while the single-filler composites show considerably higher T_d_. For AlN–BN/EP, a T_d_ of 376.1 °C is obtained. Furthermore, the AlN–BN/EP composite exhibits a residual weight of 85% at 700 °C, which is a notable improvement over 79% for AlN/EP, 53% for BN/EP, and 7% for pristine epoxy. The improved thermal performance may be ascribed to the synergistic effect of the binary filler. Good dispersibility of the hybrid fillers in the composite enhanced the polymer–filler interfacial interaction, restraining the kinetic movement of the polymer molecular chains. As a result, the thermal stability of EP composites was indirectly improved [17,25,34,53,54]. The AlN–BN/EP composite shows exceptional thermal resistance, meaning that it can successfully shield the die and bumps from thermal decomposition, which makes it a very promising electronic underfill material.

### 3.7. Electric Properties of the EP Composites

**Figure 10:**
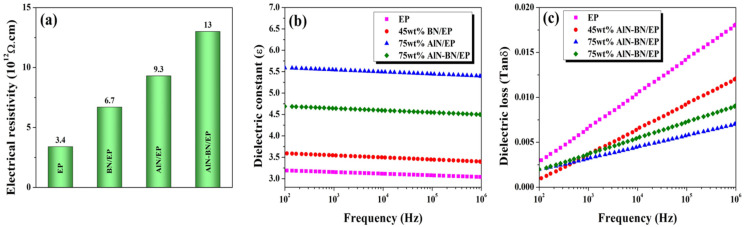
Electric properties of the as-prepared composites at the highest filler content (wt%) in this study: (**a**) volume resistivity; (**b**) dielectric constant as a function of frequency, and (**c**) dielectric loss as a function of frequency.

As illustrated in Figure 10a, the EP has outstanding insulating properties (electrical resistivity of 3.4 × 10^12^ Ω cm). Additionally, with an increase in the filler loading, the resistivity of the composites further increases, which was ascribed to the excellent electrical insulating properties of AlN and BN particles. The volume resistivity of the 75 wt% AlN–BN/EP composite is 13 × 10^12^ Ω cm, which is significantly lower than the upper resistivity threshold for electric insulators (10^8^ Ω cm) [25,67,83,84]. Therefore, the developed composites are suitable for use as IC underfill materials. The dielectric constant (ε) represents the ability to retain charge carriers in an applied electric field; in electronic packaging, ε enormously affects the signal-carrying capacity and the speed of the integrated devices; thus, a low ε value is desirable for this particular application [24,25,51,84]. As observed in Figure 10b, the ε values of both single and hybrid composites are marginally higher than that of the EP. For instance, the dielectric constant at 100 Hz is 3.19 for EP, 5.59 for AlN/EP, and 4.69 for AlN–BN/EP, which is attributed to the inherent ε values of AlN and BN particles. It can be noticed that for all composites, ε slightly decreases with an increase in frequency, which is ascribed to the dipole and Maxwell–Wagner polarizations [17,24,25,50,51,83,84,85,86,87,88,89]. According to the literature, higher AlN and BN filler loadings result in better epoxy-filler interaction, thereby increasing the interface polarization. In addition, the aforementioned polarization is induced at smaller frequencies, producing a field augmentation in the polymer matrix and therefore a variation in the total ε of the composite. Nevertheless, the overall loss in ε is not significant; ε of the composites is super-stable and low for all tested composites, suggesting that the materials possess excellent insulating properties. The dielectric loss (tan δ) represents the energy loss to heating produced during the polarity alternation of the surface of the insulating material in an applied electrical field. The tan δ values of both the single and hybrid composites are marginally smaller than those of EP, as illustrated in Figure 10c. Furthermore, the dielectric loss slightly increases with frequency, which was attributed to the discrepancy between the relaxation time of molecular dipoles and the variation in the alternating electric field in the filler and matrix [17,25,50,68,83,84,85,86,87]. Nonetheless, the interfacial interaction between the AlN–BN hybrid filler and EP restricts the movement of dipoles and the accumulation of charge carriers. Thus, the tan δ of the AlN–BN/EP composites is smaller than that of EP and is under 0.05 [50,84], indicating that the aforementioned composite is a promising IC underfill material.

## 4. Conclusions

Highly thermally conductive epoxy resin composites with hybrid fillers composed of spherical AlN particles and BN flakes were fabricated using a mechanical approach and filler surface modification. Composite viscosity was considered an essential factor in establishing the most favorable component proportions in the hybrid filler. The obtained AlN–BN/EP composites showed a TC of 10.18 W/(m K) at 75 wt% filler loading, which is approximately 46 times higher than that of the pristine epoxy. The 3D interconnected thermal conductive structure with a more effective arrangement of heat channels is responsible for the superior TC of the composites and their remarkable thermal management capability. In addition, the AlN–BN/EP composite showed improved thermal stability and a lower CTE (22.56 ppm/°C) compared to pure epoxy. In addition, high electrical resistivity, low dielectric constant, and low dielectric loss were observed for all the composites, which is a desirable result for their application. Owing to their outstanding performance, AlN–BN/EP composites have tremendous potential as underfill encapsulation materials for electronic packaging.

## Figures and Tables

**Table 1:** Detailed materials information.

Material	Mean Particle Size (μm)	TC (W/(m K))	Density (g/cm^3^)
BN	35	220	2.1
AlN	55	170	3.26
Epoxy		0.22	1.17

**Table 2:** Comparison of TC of the AlN–BN/EP composite obtained in the present study with those reported in the literature.

Fillers	Polymers	Filler Loading	TC (W/(m K))	Application	References
HoBN ^a^	PC ^b^	18.5 vol%	3.09	Thermal management materials	[18]
APTES-BNNS ^c^	Epoxy	40 wt%	5.86	Electronic packaging materials	[29]
AlN ^d^	Epoxy	20 vol%	2.26	Thermal management materials	[40]
AlN/BN	PA6 ^e^	50 vol%	1.04	Automobile industry	[53]
BN	Epoxy	50 wt%	6.09	Thermal management materials	[71]
AlN/BN	Epoxy	50 wt%	1.38	Thermal interface materials	[72]
BA-NH_2_ ^f^	CNF ^g^	50 wt%	5.93	Thermal interface materials	[73]
AlN/hBN ^h^	PTFE ^i^	30 vol%	1.04	Electronic packaging materials	[74]
AlN/BN	UHMWPE ^j^	50 wt%	7.1	Thermal management materials	[75]
AlN/BN	Epoxy	75 wt%	10.18	Electronic packaging materials	Current study

^a^ Horizontally oriented boron nitride plates. ^b^ Polycarbonate. ^c^ Boron nitride nanosheets functionalized with (3-aminopropyl) triethoxysilane. ^d^ Nanoflower-like AlN. ^e^ Polyamide 6. ^f^ Amine-group-treated hybrid filler comprising boron nitride and aluminum nitride. ^g^ Cellulose nanofiber. ^h^ Spherical-like aluminum nitride particles and hexagonal boron nitride platelets. ^i^ Polytetrafluoroethylene. ^j^ Ultrahigh-molecular-weight polyethylene.

## Data Availability

Not applicable.

## Supplementary Materials

The following supporting information can be downloaded at: https://www.mdpi.com/article/10.3390/polym14142950/s1, a video showing a demonstration of the infrared thermal images of the hybrid composites (MP4).
